# Non-invasive genetic monitoring for the threatened valley elderberry longhorn beetle

**DOI:** 10.1371/journal.pone.0227333

**Published:** 2020-01-17

**Authors:** Raman P. Nagarajan, Alisha Goodbla, Emily Graves, Melinda Baerwald, Marcel Holyoak, Andrea Schreier

**Affiliations:** 1 Department of Animal Science, University of California Davis, Davis, CA, United States of America; 2 Department of Environmental Science and Policy, University of California Davis, Davis, CA, United States of America; Lund University, SWEDEN

## Abstract

The valley elderberry longhorn beetle (VELB), *Desmocerus californicus dimorphus* (Coleoptera: Cerambycidae), is a federally threatened subspecies endemic to the Central Valley of California. The VELB range partially overlaps with that of its morphologically similar sister taxon, the California elderberry longhorn beetle (CELB), *Desmocerus californicus californicus* (Coleoptera: Cerambycidae). Current surveying methods are limited to visual identification of larval exit holes in the VELB/CELB host plant, elderberry (*Sambucus* spp.), into which larvae bore and excavate feeding galleries. Unbiased genetic approaches could provide a much-needed complementary approach that has more precision than relying on visual inspection of exit holes. In this study we developed a DNA sequencing-based method for indirect detection of VELB/CELB from frass (insect fecal matter), which can be easily and non-invasively collected from exit holes. Frass samples were collected from 37 locations and the 12S and 16S mitochondrial genes were partially sequenced using nested PCR amplification. Three frass-derived sequences showed 100% sequence identity to VELB/CELB barcode references from museum specimens sequenced for this study. Database queries of frass-derived sequences also revealed high similarity to common occupants of old VELB feeding galleries, including earwigs, flies, and other beetles. Overall, this non-invasive approach is a first step towards a genetic assay that could augment existing VELB monitoring and accurately discriminate between VELB, CELB, and other insects. Furthermore, a phylogenetic analysis of 12S and 16S data from museum specimens revealed evidence for the existence of a previously unrecognized, genetically distinct CELB subpopulation in southern California.

## Introduction

**Fig 1:**
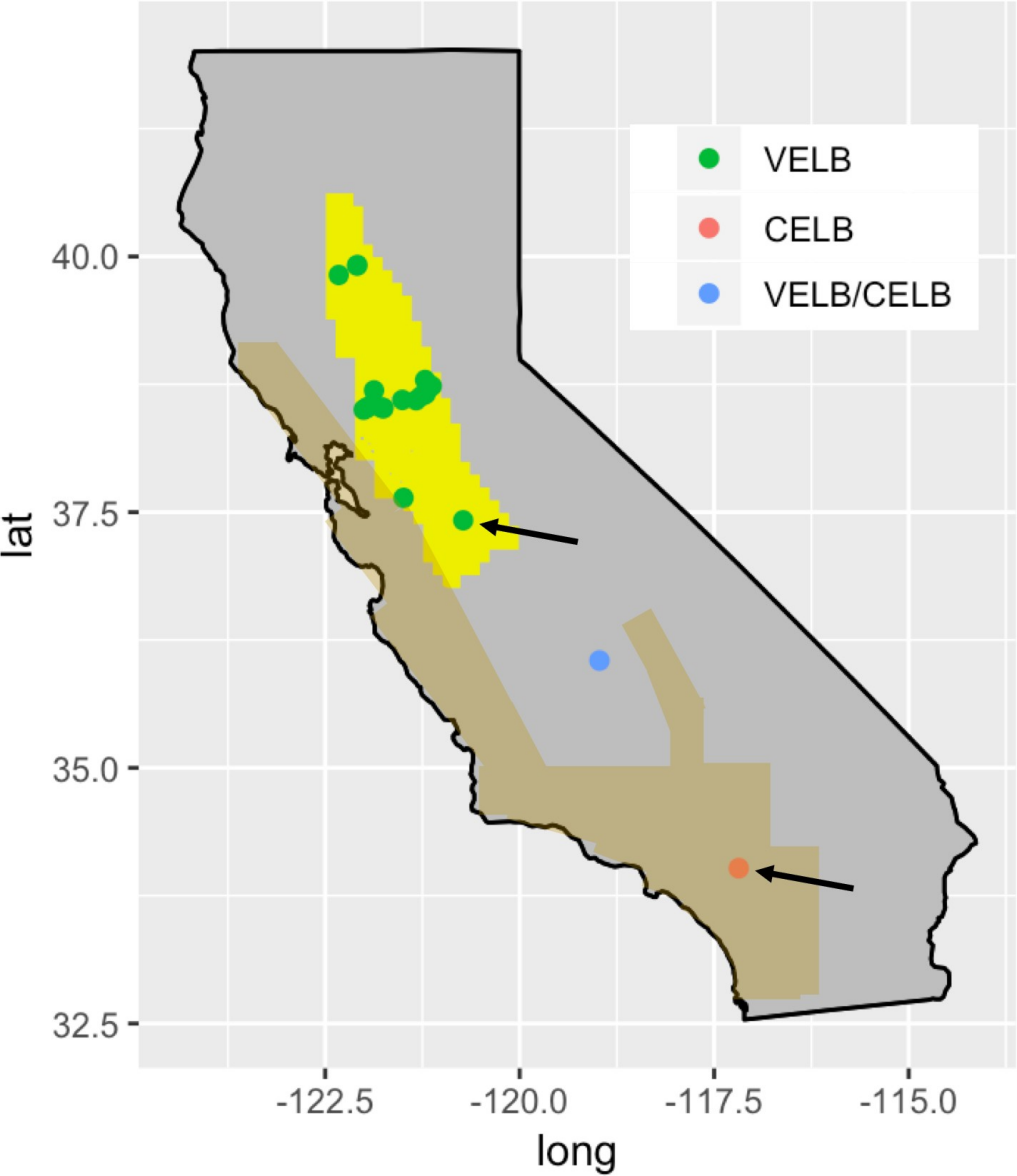
Map of VELB and CELB ranges within California. Individual points show frass collection locations that yielded sequencing data. One frass sample that was collected in the southern Central Valley outside of the VELB and CELB official ranges is labeled “VELB/CELB” to indicate uncertainty about which subspecies might be present. Arrows show locations where frass-derived sequences matched VELB or CELB museum specimens collected from nearby locations.

The valley elderberry longhorn beetle (VELB) is a wood-boring subspecies of beetle of the Cerambycidae found only in the Central Valley of California, U.S.A. [1] (Fig 1). The subspecies is sexually dimorphic, with males displaying red-orange elytra (wing covers) with four dark elongate spots, and females showing dark, metallic green to black elytra with bright red-orange borders [2]. VELB inhabit elderberry shrubs (*Sambucus* spp.) for nearly their entire life cycle, and are commonly found in riparian forests and adjacent uplands near Central Valley waterways [3,4]. After hatching from eggs laid externally on leaves and stems, VELB larvae bore into, feed on the pithy center, and pupate within elderberry stems, culminating with the emergence of adults through distinctive exit holes [5]. During their 1–2 year inhabitation of the stems, VELB excavate feeding galleries and deposit fecal material mixed with wood shavings (frass) that remain after adults have exited the hole [5–7]. After emergence, adult males can live up to 5 days under laboratory conditions, whereas adult females live up to 3 weeks [8].

Due to declining populations resulting from habitat loss, in 1980 VELB was listed as threatened under the U.S. Endangered Species Act [9]. The Central Valley of California has been extensively modified by farming, urbanization, and flood control measures, diminishing and altering the riparian habitats critical for VELB [10,11]. A proposal to delist the subspecies was made in 2012 [12] but ultimately withdrawn by the U.S. Fish and Wildlife Service after external review [11,13]. VELB continues to be threatened by habitat fragmentation and loss, climate change, predators, pesticides and invasive species [11,14].

Despite its conservation significance, VELB has been historically difficult to survey and study. VELB population densities are low, and field surveys suffer from low probability of detection [4,15]. Live adult VELB are infrequently encountered in the wild [2,4,7], and occurrence records are mostly based on observation of exit holes in elderberry stems, which themselves occur at low density in elderberry stands. Exit hole surveys are non-invasive but can be inaccurate due to misidentification of holes produced by other xylophagous beetles or wasps [16]. The degree of misidentification of holes is not known. When adult VELB are (rarely) encountered, VELB females are indistinguishable from a closely related subspecies, the California elderberry longhorn beetle (CELB). CELB females are morphologically similar to VELB females, but CELB males have dark elytra bordered with a thin red margin and thus are distinguishable from VELB males [2]. In our experience in the field, the exit holes produced by VELB and CELB are indistinguishable, as might be expected with two closely related members of the same species that share many morphological features as well as life history and biology [17,18]. The CELB is not listed as threatened or endangered, although the subspecies is less studied than the VELB. CELB inhabit coastal California, parts of the Sierra Nevada, and the central and southern San Joaquin Valley and southwards, including the highly urbanized Los Angeles and San Diego areas [2,19,20] (Fig 1). VELB and CELB ranges overlap along the eastern edge of the Coast Range, and intermediate forms have been observed [2,8,16]. The exit holes of VELB cannot be distinguished from those of CELB, and in areas of overlap, exit hole survey methods are subject to ambiguity about which subspecies is present. For these reasons, there is a pressing need for an accurate survey tool that could improve the probability of detection of VELB and identify subspecies in the absence of adult specimens. A non-invasive survey technique that minimizes mortality and interference with natural VELB behaviors would be particularly useful.

Genetic barcoding of non-invasively collected DNA has been used to rapidly survey populations of many taxa including invertebrates (e.g. [21–24]). Species-specific DNA fragments can be used to document species presence in soil samples [25], gut contents [26,27], or fecal material [28]. Insect frass has been shown to be a viable source of non-invasive DNA sampling for butterfly caterpillars [29], scarab beetles [30], and bumblebees [31]. An indirect, genetic method could provide a much-needed complementary approach to existing exit hole survey methods for VELB and CELB. Frass would be an ideal source of material, since it is readily obtainable from unoccupied exit holes with minimal disturbance. In this study we generated VELB and CELB barcode reference sequences for multiple loci from a panel of museum voucher specimens. In parallel, we collected frass from exit holes at multiple locations and used nested PCR and direct Sanger sequencing to test the ability of indirect molecular techniques to identify VELB and CELB. In addition, phylogenetic analysis of VELB/CELB museum specimens identified a previously unknown, genetically distinct population of CELB in southern California.

## Materials and methods

### Non-destructive DNA extraction, PCR and sequencing of VELB and CELB museum specimens

Non-destructive DNA extraction involved full immersion of specimens in digestion buffer containing 3 mM CaCl_2_, 2% sodium dodecyl sulfate (SDS), 40 mM dithiotreitol (DTT), 250 mg/ml proteinase K, 100 mM Tris buffer pH 8 and 100 mM NaCl and incubated overnight at 55°C with gentle agitation. The specimens were then removed, immersed in 100% ethanol and air-dried. DNA was purified from the digestion buffer with the QIAquick PCR purification kit (Qiagen) following the manufacturer’s protocol. DNA concentrations were determined using a Qubit Fluorimeter along with the Broad Range dsDNA Assay Kit (ThermoFisher). Samples were normalized to a working concentration of 10 ng/μl and amplified in PCR reactions using the primers shown in S1 Table. PCR reactions were performed in a total volume of 25 μl with the following components: 1X TaKaRa Buffer, 1.4 μM MgCl_2_ (Roche), 0.4 μM dNTPs (TaKaRa), 0.4 μM of each primer, 0.2 μl TaKaRa Ex Taq, and 1 μl of template DNA. (For DNA concentrations less than 10 ng/μl, we used 2 μl of undiluted DNA). After initial denaturation at 94°C for 3 minutes, 35 cycles were performed of 94°C for 30s, 55°C for 60s, 72°C 60s. Final extension was 10 minutes at 72°C. PCR amplifications were visualized with 2% agarose electrophoresis, and positive PCR amplicons were purified with Ampure beads (or with gel extraction (Qiagen) if necessary) and submitted for Sanger sequencing to Quintara Biosciences. Sequencing data were analyzed with Sequencher 4.8 and alignments were made and trimmed in MEGA7 [32] using the Clustal W approach with default settings.

### Field survey and sampling protocol

Thirty-six sites were surveyed for VELB or CELB in 2016–2018. We surveyed known sites within the VELB range, based on the presence of VELB in past surveys [4,5] and accessibility to the public. We deliberately selected sites to include the core area of the VELB range, suspected hybrid zones with CELB (the southern Central Valley or south of Napa Valley), and some areas known to be inhabited by CELB. At each site, all elderberry shrubs that could be visually detected from marked trails and access points were inspected for larval exit holes. Exit holes were classified as either new (beetle emergence within the current year) or old (beetle emergence prior to the current year). New holes were those that appeared light-colored inside and had not yet begun to heal if in live stems. Surveying consisted of visual scanning of main stems and branches for exit holes, and recording the numbers of exit holes found. When an exit hole was found, tweezers were used to extract any material possible from inside the elderberry stem. Frass/feces and/or exuviae were collected from each exit hole and placed into a clean plastic Eppendorf tube. When multiple exit holes were detected on the same elderberry shrub, material from each exit hole was placed in a separate tube. When an exit hole was detected in a dead stem, the stem was collected when possible and brought back to the lab and broken apart to extract any exuviae and/or frass/feces. Live stems were not collected and were only sampled by removing frass/feces non-destructively. Upon returning from the field, samples were stored in a -20°C freezer until later processing. As our surveying did not result in any incidental take of either subspecies, since it was completely limited to collecting frass, no collecting permit was required. Nearly all of the locations that we sampled were public access and did not require any permissions. The only exceptions are sites listed as "private" for which we had explicit permission to access through our personal relationships with the landowners (contingent upon the locations remaining anonymous). We obtained permission to access the Russell Ranch site (UC Davis property) from the manager via email.

### DNA extraction, PCR, and sequencing of frass samples

DNA extractions for the majority of frass samples were performed with the DNeasy PowerSoil Pro Kit (Qiagen) following the manufacturer’s protocol with the minor adjustment of eluting the DNA in 60 μl of the provided elution buffer pre-warmed to 56°C. DNA concentrations were determined using a Qubit Fluorimeter along with the Broad Range dsDNA Assay Kit (ThermoFisher). Samples were normalized to a working concentration of 10 ng/μl and 2 μl of each was used in PCR reactions. PCR primers used in this study are shown in S1 Table. PCR reaction setup and cycling were the same as described above, but with 2 μl template per reaction. For nested PCR, 2 μl of a 1:100 dilution of the first PCR product was used as the template for the second PCR. Amplification products were checked by 2% agarose gel electrophoresis, and PCR amplicons were purified with Ampure beads (or with Qiagen gel extraction if multiple bands were observed) and submitted for sequencing at Quintara Biosciences. Sequencing data were analyzed with Sequencher 4.8 and alignments were visualized with MEGA 7. To determine the species from which a frass specimen originated, frass sequences were queried with Basic Local Alignment Search Tool–Nucleotide **(**BLASTN) and compared to 12S and 16S rRNA alignments from VELB/CELB museum samples.

### Phylogenetic tree methods

For phylogenetic tree construction, we selected 12S and 16S rRNA genes because they amplified consistently across samples. We calculated the number of transitions and transversions and nucleotide composition and used these metrics to parameterize construction of neighbor-joining trees in MEGA7 [33]. The 12S and 16S genes were concatenated before tree construction for all individuals that contained complete sequence at those genes (18 VELB, 18 CELB). Gaps and missing data were deleted and uniform evolutionary rates among sites and lineages was assumed. The only gap present was at 126–128 bp in 12S. (Analysis was performed with and without gap removal and the same topology was observed.) Trees were constructed using the Tamura 3-parameter model [34] and tested using 1000 bootstrap replicates [35].

## Results

To generate reference barcode sequences for frass genetic identification, we first obtained VELB and CELB dry specimens from multiple museum collections. The specimens were collected across a wide geographic area, encompassing 22 California counties (S2 Table). To preserve these valuable specimens, we utilized a non-destructive DNA extraction protocol based on Thomsen and coworkers [36] to extract DNA from 44 specimens (19 VELB and 25 CELB), including those collected as far back as 1914 (S1 Fig). We tested 27 primer pairs targeting barcoding loci, several of which were developed for this study (S1 and S3 Tables). Fourteen primer pairs yielded successful PCR amplification for at least a subset of museum samples, and eight of these generated clean sequencing data (S3 Table). These data were from the following genes: cytochrome c oxidase 1 (COI, mitochondrial, 1 primer pair); 18S rRNA (nuclear, 2 primer pairs); 16S rRNA (mitochondrial, 2 primer pairs); and 12S rRNA (mitochondrial, 3 primer pairs). All sequences were deposited to GenBank (S4 Table). Sequences from all samples were aligned and inspected for the presence of variable sites. For COI and 18S, none were found. In contrast, multiple variable sites were identified for amplicons in the 12S and 16S genes (S5 Table). We searched these sequences for potential subspecies-specific SNPs that would enable discrimination between VELB and CELB, but none were found at these loci (S3 and S5 Tables).

**Fig 2:**
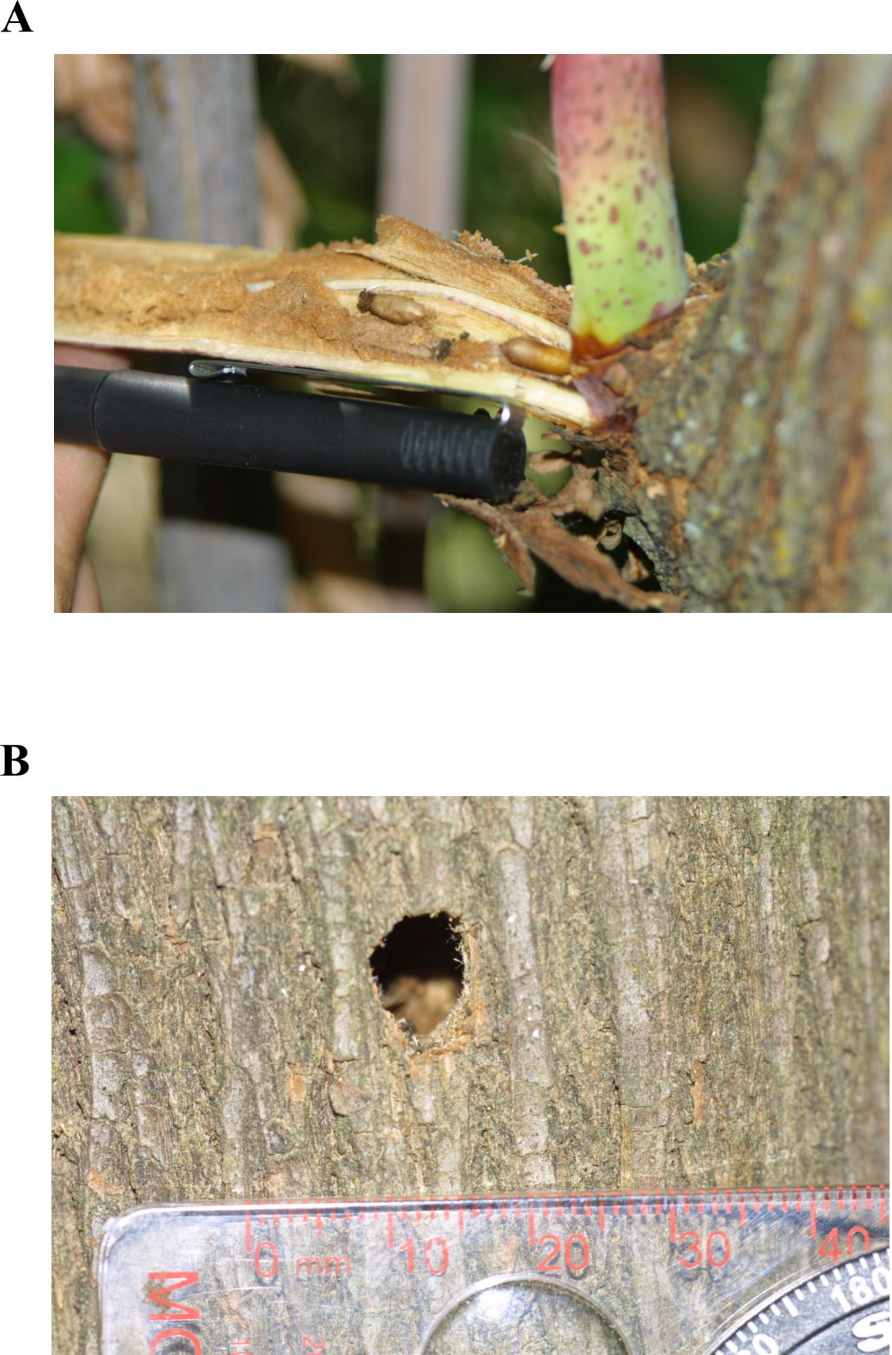
VELB exit holes in elderberry shrubs. A. Archive photo from 1997 showing VELB pupae and frass in a live elderberry stem. B. New (current year) exit hole in elderberry stem from field survey/sampling performed during this study.

To test the possibility of genetic detection from frass, samples were collected from elderberry exit holes in conjunction with VELB field survey work [37] (Fig 2A and 2B). Across these survey sites, 535 individual shrubs were examined for VELB/CELB exit holes (Table 1). Of the 212 total exit holes detected, 118 were old holes (beetle emergence occurred in a previous year) while 34 holes were determined to be new (beetle emergence occurred earlier in the current season). Additional CELB frass samples were collected separately in southern California by a consulting company. Altogether, 84 frass samples were collected.

**Table 1:** Exit hole survey and frass collection.

Site	County	Shrubs surveyed	Total exit holes	Old holes	New holes	Unknown holes	Samples collected
Folsom Lake-Peninsula Campground	El Dorado	5	2	2	0	0	4
ARP-River Bend Park	Sacramento	33	2*	0	0	0	2
McConnell SRA	Merced	14	5*	0	0	0	6
George J. Hatfield SRA	Merced	12	1*	0	0	0	0
ARP-Discovery Park	Sacramento	75	24*	0	0	0	9
Bobelaine Audubon Sanctuary	Sutter	28	0	0	0	0	0
Turtle Bay Exploration Park	Shasta	28	0	0	0	0	0
Anderson River Park	Shasta	5	0	0	0	0	0
Reading Island SRA	Shasta	17	0	0	0	0	0
Russell Ranch—VELB Mitigation Area	Yolo	62	2*	0	0	0	0
Russell Ranch	Yolo	13	7*	0	0	0	6
Putah Creek—Raptor Center	Yolo	18	3*	0	0	0	2
Putah Creek—Riparian Reserve	Yolo	14	8*	0	0	0	3
Putah Creek—Pedrick Road	Yolo	7	0	0	0	0	0
Putah Creek—Winters (private)	Yolo	21	13	3	10	0	13
Lake Solano Park	Solano	22	7	6	1	0	4
Folsom Lake-Mississippi Bar	Sacramento	12	10	7	3	0	3
ARP-Upper Sunrise Area Boat Launch	Sacramento	19	20	19	1	0	2
Woodson Bridge SRA	Tehama	15	16	10	6	0	2
Tehema County River Park	Tehama	6	2	1	1	0	1
Big Chico Creek Access	Butte	5	0	0	0	0	0
Putah Creek—Solano County (private)	Solano	26	30	22	8	0	6
American River Parkway-Cal Expo	Sacramento	0	0	0	0	0	0
Yaudanchi Ecological Reserve	Tulare	14	33	31	2	0	2
Cache Creek Nature Preserve	Yolo	24	4	3	1	0	2
Putah Creek Winters	Yolo	10	7	1	4	2	5
Sierra College Nature Trail	Placer	3	5	4	0	1	1
Black Butte Rec Area—Low. Stoney Creek	Tehama	3	1	0	0	1	1
Black Butte Rec Area—Buckhorn	Tehama	0	0	0	0	0	0
Black Butte Rec Area -Big Oak Trail	Glenn	3	0	0	0	0	0
Black Butte Rec Area—Grizzly Flat	Tehama	2	0	0	0	0	0
Kern River Preserve	Kern	12	1	0	0	1	0
Henry Cowell State Park	Santa Cruz	0	0	0	0	0	0
Fall Creek Unit	Santa Cruz	0	0	0	0	0	0
Wilder Ranch	Santa Cruz	7	3	3	0	0	0
Corral Hollow Ecological Reserve	San Joaquin	unknown	6	6	0	0	6
** **	**Totals:**	**535**	**212**	**118**	**37**	**5**	**80**

Summary of new holes (beetle emergence in the current year) and older holes (beetle emergence in a previous year). CELB frass was also collected from an additional location in southern California (not listed here).

*Exit hole age was not assessed.

DNA extractions from frass samples yielded DNA concentrations ranging from 1–580 ng/μl, with an average concentration of 63 ng/μl. DNA was extracted from 84 samples, and a subset of these was checked for DNA quality by agarose gel electrophoresis. All samples examined showed mostly intact DNA, with relatively minor degradation, by agarose gel electrophoresis (S2 Fig).

Single band PCR products were successfully amplified from 23 frass samples and 26 sequences were queried using BLASTN (Table 2). The top BLASTN hits (all > 80% identity) for all 26 sequences corresponded to insects, including other beetles, earwigs, flies, and other insects. Since neither VELB nor CELB 12SS or 16S sequences were present in the GenBank database during the query, none of the top BLASTN hits corresponded to VELB or CELB. However, three 12S top hits were from other longhorn beetles (from the same taxonomic family, Cerambycidae, as VELB and CELB). We found that for these three samples, the 12S sequences exactly matched sequences from VELB or CELB museum specimens (Table 2 and S3 Fig). Furthermore, the frass sequences were identical to museum specimens collected at nearby locations. One frass specimen collected from the southern portion of the VELB range showed 100% sequence identity with VELB/CELB museum specimens from the VELB and northern CELB ranges (Fig 1, top arrow and S3 Fig). Two frass specimens collected approximately 300 meters from one another in San Bernardino county in southern California, in the southern part of the CELB range, showed 100% sequence identity with CELB museum specimens collected from southern California (Fig 1, bottom arrow and S3 Fig).

**Table 2:** Top BLASTN hits for 12S and 16S rRNA frass-derived sequences.

Gene	Sample	Hole age	Top BLASTN hit	Query cover	E value	Identity	Common name
12S	4*	new	Arhopalus rusticus	0.95	0	0.92	species of longhorn beetle
	14	new	Sinacidia flexuosa	0.84	0	0.9	In family Tephritidae-Fruit Flies
	15	old	Sinacidia flexuosa	0.81	0	0.9	In family Tephritidae-Fruit Flies
	16	old	Accanthopus velikensis	1	0	0.9	species of darkling beetle
	17	new	Matsumurania sapporensis	0.95	0	0.9	In family Tephritidae-Fruit Flies
	18	new	Megaselia impariseta	1	0	1	Common Humpbacked fly
	20	old	Megaselia impariseta	1	0	0.97	Common Humpbacked fly
	26	new	Cardiothorax howitti	0.94	0	0.9	species of darkling beetle
	34	new	Nalassus dryadophilus	1	0	0.98	species of darkling beetle
	35	old	Promethis angulata	0.94	0	0.89	species of darkling beetle
	39	new	Tachinus subterraneus	0.91	0	0.97	species of Rove Beetle
	46	old	Hypera brunneipennis	1	0	1	Egyptian Alfalfa Weevil
	47	old	Olophrum piceum	0.79	0	0.86	species of Rove Beetle
	48	new	Tachinus subterraneus	0.92	0	0.95	species of Rove Beetle
	55	new	Laparocerus prainha	1	0	0.87	species flightless weevil
	66	new	Triarthria setipennis	1	0	0.97	species of tachinid fly
	70	new	Staphylinidae sp	1	0	0.92	species of Rove Beetle
	72	new	Accanthopus velikensis	1	0	0.9	species of darkling beetle
	73	new	Dryocoetes autographus	0.97	0	0.89	species of bark beetle or weevil
	F02A*	unk	Strangalia luteicornis	1	0	0.9	species of flower longhorn beetle
	F03	unk	Minettia nigriventris	0.97	0	0.93	family Lauxaniidae—small fllies
	F04A*	unk	Strangalia luteicornis	1	0	0.89	species of flower longhorn beetle
16S	4	new	Irbisia pacifica	0.99	0	0.99	Pacific grass bug
	17_For	new	Forficula auricularia	1	0	0.97	European earwig
	17_Rev	new	Anthrax proconcisus	0.99	0	0.85	Bee fly
	18	new	Forcipomyia fuliginosa	0.98	0	0.84	Biting midge
	22	new	Forcipomyia hygrophila	1	0	0.85	Biting midge

Twenty-six PCR products yielded analyzable sequences (22 from 12S and 4 from 16S). These were queried for database matches using BLASTN. For samples with database matches (alignment > 80% identity), the top BLASTN hit is shown. The three sequences that have 100% matches to VELB/CELB museum specimens are bolded and labeled with an asterix. Unk., unknown.

**Fig 3:**
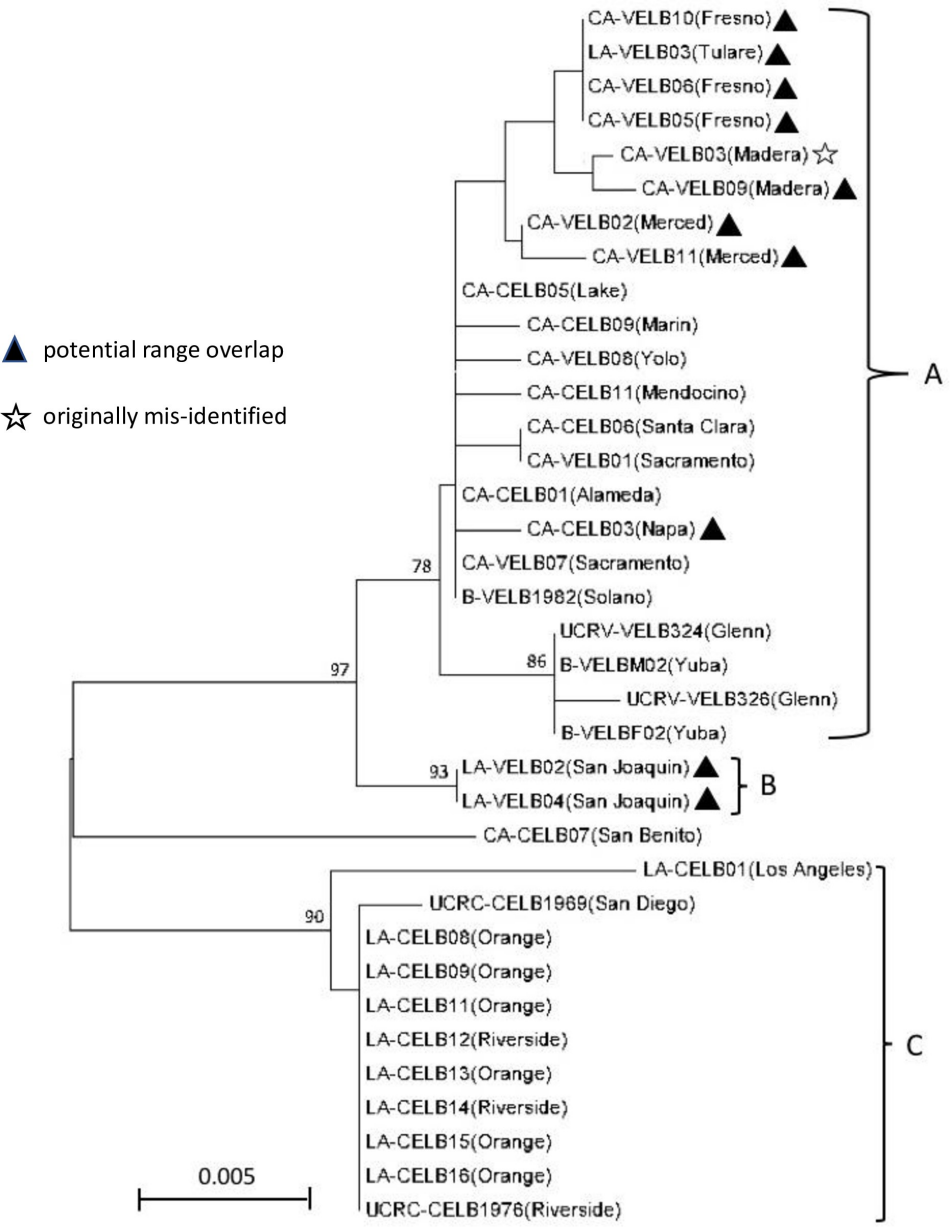
Neighbor-Joining tree constructed from 514 base pairs of the 12S and 16S rRNA genes. The optimal tree with the sum of branch lengths = 0.071 is shown. Three monophyletic clades are labeled “A,” “B,” and “C.” Bootstrap values for highly supported nodes (>75%) are shown above the branches. Nodes without values indicate poor (<75%) bootstrap support. County from which each sample was collected is in parentheses, triangles indicate samples from areas of potential range overlap, and a star indicates a museum specimen initially misidentified as CELB.

To further understand the genetic relationships across VELB and CELB populations, we used the concatenated 12S and 16S sequences from the museum specimens to construct a neighbor-joining phylogenetic tree (Fig 3). We identified 21 variable sites across 277 base pairs of the 12S rRNA sequence data, after removal of a three base pair gap in 18 VELB and 23 CELB samples. Nucleotide composition was A/T biased, with bases 41.9–43.0% T, 38.6–40.1% A, 11.2–12.6% C, and 6.1–6.9% G. Fifteen transitions and six transversions were identified. In the 16S rRNA sequence data, 13 variable sites were identified in 20 VELB and 21 CELB samples across 237 bases, with no gaps or missing data. Nucleotide composition at this locus was A/T biased, with bases 43.5–45.1% T, 39.2–40.9% A, 9.7–11.4% G, and 5.1–5.5% C. Eight transversions and five transitions were identified. Two sites were highly variable, with three (tri-allelic) and four (quad-allelic) different nucleotides detected.

Three highly supported clades were identified in the neighbor-joining tree constructed using the Tamura 3-parameter model [34] (Fig 3). Clade A contained most VELB specimens and 33% of CELB specimens, all originating from northern California collection locations. All sub-branches within Clade A had relatively low bootstrap support, indicating statistically uncertain topology within this branch. Clade B consisted of the two remaining VELB that were collected from San Joaquin county, a location of potential range overlap for the two subspecies. Clade C included only CELB specimens originating from the southernmost part of that subspecies’ range.

## Discussion

Insect frass has emerged as a non-invasive DNA source for multiple types of genetic studies, including those of beetles. This has included studies of pathogen infection of both beetles and their host plants [38] and dietary analysis [39]. Frass has also been utilized for molecular species identification, which can be indispensable in distinguishing closely related species [30]. In addition, frass genetic methods can reveal gender and identify transgenic individuals, even in visually similar early larval stage insects [40]. A PCR-based approach has been documented for invasive wood-boring insects [41] but there is also potential for non-lethal genetic sampling of rare and protected organisms. VELB and CELB frass could provide a source of DNA from these beetles, although the quality and quantity of DNA *in situ* is not currently known and might be variable. However, frass collection has the advantage that it does not interfere with or harm VELB adult beetles, which are protected by law and might be sensitive to disturbance.

Several factors may affect the efficiency of detecting VELB/CELB DNA in frass. First, some of the exit holes from which frass was collected could have been created by other xylophagous beetles or insects, and were never occupied by VELB/CELB. This limitation of the exit hole survey method has been previously documented [16] and underscores that a genetic detection method could improve the accuracy of visual surveying methods. Second, even if the exit hole was created by VELB/CELB, we hypothesize that some of these frass samples contain an admixture of DNA from multiple insects, potentially at varying proportions from sample to sample. It is possible that some of the older holes are created by VELB/CELB and leave behind feces but then other insects move into the holes and deposit fresher feces or exuviae (cast-off outer skin) with more intact DNA in the frass sample. We frequently observed other insects in or around the exit holes, and earwigs were particularly common around older holes. Earwigs are insect predators and their prey include beetles. Finally, any VELB/CELB DNA will degrade over time, likely decreasing its probability of detection in older samples. Additionally, DNA from microorganisms and from the elderberry plant itself might be present at much higher concentrations than VELB/CELB DNA or DNA from other insects, further hindering detection.

Our data support effects from all of these factors. Forty-three (of 84 total) frass samples could not be amplified by PCR, suggesting low VELB/CELB DNA concentrations or DNA degradation. PCR inhibitors could also contribute to this (see below). When PCR and sequencing were successful, all of the top BLAST hits were insects, consistent with exit hole creation and/or occupancy by multiple types of insects. These included earwigs, flies, midges, and other beetles. For future studies, cloning of individual PCR amplicons or deep sequencing of the admixed PCR product would allow a more granular and comprehensive picture of the organisms present (at least for sequences that can be PCR amplified). The frass samples might also contain PCR inhibitors from elderberry wood shavings, polysaccharides from feces, or other materials [42,43]. The extraction method we used does include a PCR inhibitor removal step, but this could potentially be further optimized. Finally, some mitochondrial loci might be more challenging to amplify from frass than others. Similar to another publication [40], we were unsuccessful in amplifying COI in our beetle frass samples, although these primers successfully amplified a subset of museum specimens (S3 Table).

Despite these challenges, sequences generated from three frass samples exactly matched references obtained from VELB/CELB museum specimens, strongly suggesting indirect genetic detection. One frass-derived sequence from a northern California collection location matched multiple museum specimens from clade A in the phylogenetic analysis, and frass collected from southern California matched museum specimens in clade C (Fig 1 and S3 Fig). Furthermore, the frass data, although limited, are consistent with the phylogenetic analysis from museum specimens pointing towards a genetically distinct CELB population in southern California. Both the museum and frass samples from this putative subpopulation were all collected from sites south of the Transverse Mountain Range in Southern California, which runs east to west and might present a geographic barrier promoting allopatric differentiation. Alternatively, the observed branching pattern could be due to decreased hybridization at the southernmost end of the CELB range, which is further from the VELB range. Additional genetic data, either from non-invasively collected frass, or high-quality DNA extracted from fresh specimens, would help to conclusively determine the true taxonomic relationships among these members of the Cerambycidae. Although there is scant published genetic data about either subspecies, a doctoral thesis that performed molecular taxonomic analysis of multiple *Desmocerus* species found a relatively large genetic distance between VELB and CELB clades, such that the data “might appear to suggest that the two populations are easily separable as distinct species” [44]. Notably, the three VELB specimens used in that study were from Ord Bend (Northern CA) and the two CELB were from San Bernardino (southern CA), which in our phylogeny would correspond to clades A and C, respectively. The author of that study was careful to emphasize the limitations of the data, and declined to suggest any changes to the existing VELB/CELB taxonomy. Similarly, although we have interrogated more samples across a broader geographic range, our phylogenetic data should also be considered preliminary and interpreted with caution. Additional samples combined with a genome-scale approach, such as RADseq [45] would likely allow more definitive taxonomic conclusions. Since a comprehensive genetic analysis of VELB and CELB has been hindered to date due to the lack of fresh specimens for DNA extraction, one potentially productive approach involves pheromone-based trapping of live beetles, using the recently discovered attractant sex pheromone (*R*)-Desmolactone [46,47]. Such an approach would enable efficient collection of fresh VELB and CELB samples from multiple, specific locations across their core ranges, and can be stopped at each site when sufficient sample numbers have been collected, minimizing the number of individuals removed from potentially sensitive populations. The application of new approaches, both for sample collection as well as genetic analysis, will afford a much deeper and complete understanding of these elusive and threatened beetles.

## Supporting information

S1 Table PCR primers used in this study. Mitochondrial and nuclear DNA regions were screened in VELB and CELB museum samples to identify diagnostic loci, and nested primers for mitochondrial 12S and 16S genes were used to amplify DNA extracted from frass material.
(XLSX)

S2 Table Collection information for VELB and CELB beetle samples used in genetic analyses. (XLSX)

S3 Table PCR and sequencing results for 27 tested primer sets on museum specimens. (XLSX)

S4 Table GenBank Accession numbers for VELB and CELB museum specimens. (XLSX)

S5 Table Highly variable sites (SNPs) in the mitochondrial 12S and 16S rRNA genes across VELB and CELB museum specimens. Based on the original geographical collection location, all specimens were annotated either high or low confidence (column 3). High confidence samples are those collected from core regions of the known range for each subspecies and are less likely to be subspecies or hybrids.
(XLSX)

S1 Fig Two VELB and two CELB beetle specimens from the University of California Riverside's Entomology Research Museum. Left, pre-extraction; right, post-extraction. Use of a non-destructive DNA isolation protocol allowed for successful DNA extraction without morphological damage.
(PDF)

S2 Fig Frass DNA extractions (samples numbered 1–24) from the Qiagen PowerSoil Pro kit. Purified DNAs were run on a 1% agarose gel to examine DNA quality.
(PDF)

S3 Fig Multiple sequence alignment showing the exact DNA match between frass samples and multiple museum specimens. Frass sample 4 showed a 100% match to multiple museums specimens that had been assigned to clade A (from the 12S/16S phylogenetic tree in Fig 2). In addition, Frass sample 4 showed a 100% match to the two museum specimens comprising clade B (not shown). Frass samples F02A and F04A both showed 100% sequence identity to specimens assigned to clade C.
(PDF)
