# Supplementary figures and images for: Non-invasive genetic monitoring for the threatened valley elderberry longhorn beetle
Source: PLoS One. 2020 Jan 17;15(1):e0227333. doi: 10.1371/journal.pone.0227333 (PMC6968946; doi:10.1371/journal.pone.0227333)

**S1 Figure**

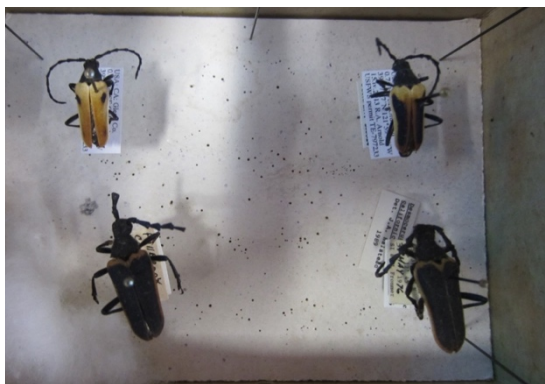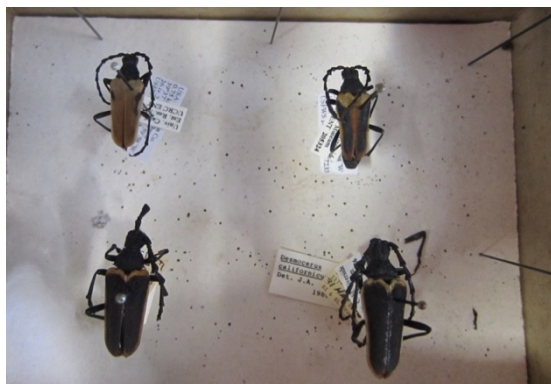

Supplement: S1 Fig — Left, pre-extraction; right, post-extraction. Use of a non-destructive DNA isolation protocol allowed for successful DNA extraction without morphological damage. (PDF) [file pone.0227333.s006.pdf]

S2 Figure

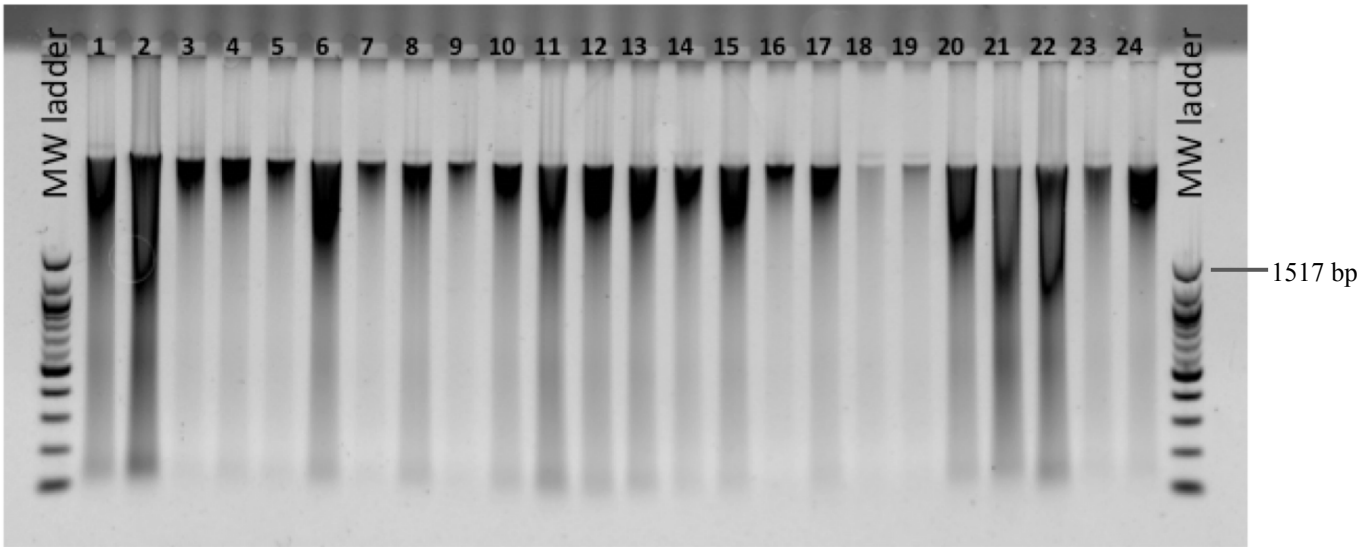

Supplement: S2 Fig — Purified DNAs were run on a 1% agarose gel to examine DNA quality. (PDF) [file pone.0227333.s007.pdf]

S3 Figure.

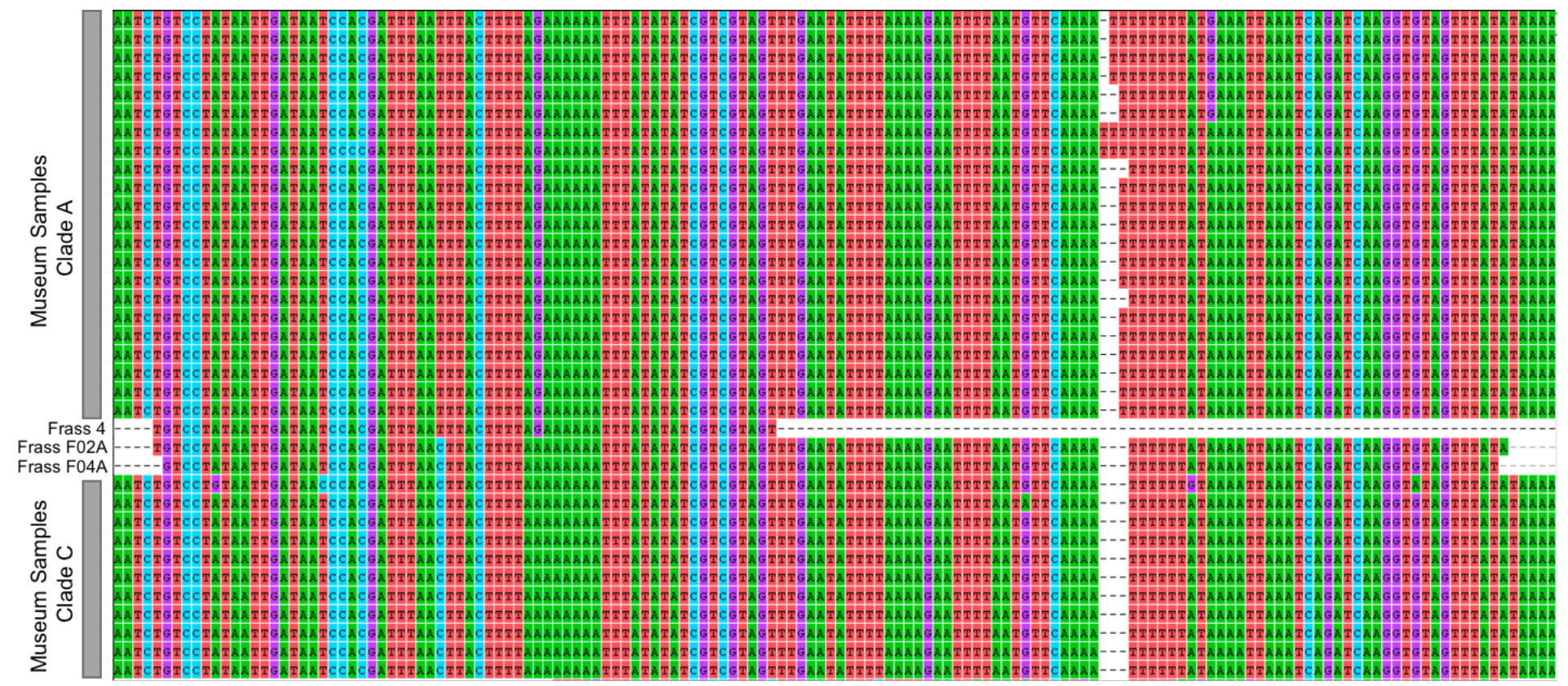

Supplement: S3 Fig — Frass sample 4 showed a 100% match to multiple museums specimens that had been assigned to clade A (from the 12S/16S phylogenetic tree in Fig 2). In addition, Frass sample 4 showed a 100% match to the two museum specimens comprising clade B (not shown). Frass samples F02A and F04A both showed 100% sequence identity to specimens assigned to clade C. (PDF) [file pone.0227333.s008.pdf]
